# Multichromosomal Mitochondrial Genome of *Paphiopedilum micranthum*: Compact and Fragmented Genome, and Rampant Intracellular Gene Transfer

**DOI:** 10.3390/ijms24043976

**Published:** 2023-02-16

**Authors:** Jia-Xing Yang, Nicolas Dierckxsens, Ming-Zhu Bai, Yan-Yan Guo

**Affiliations:** 1 College of Plant Protection, Henan Agricultural University, Zhengzhou 450046, China; 2 Center for Human Genetics, KU Leuven, 3000 Leuven, Belgium

**Keywords:** Orchidaceae, slipper orchid, hybrid assembly, multichromosomal genome, intracellular gene transfer

## Abstract

Orchidaceae is one of the largest families of angiosperms. Considering the large number of species in this family and its symbiotic relationship with fungi, Orchidaceae provide an ideal model to study the evolution of plant mitogenomes. However, to date, there is only one draft mitochondrial genome of this family available. Here, we present a fully assembled and annotated sequence of the mitochondrial genome (mitogenome) of *Paphiopedilum micranthum*, a species with high economic and ornamental value. The mitogenome of *P. micranthum* was 447,368 bp in length and comprised 26 circular subgenomes ranging in size from 5973 bp to 32,281 bp. The genome encoded for 39 mitochondrial-origin, protein-coding genes; 16 tRNAs (three of plastome origin); three rRNAs; and 16 ORFs, while *rpl10* and *sdh3* were lost from the mitogenome. Moreover, interorganellar DNA transfer was identified in 14 of the 26 chromosomes. These plastid-derived DNA fragments represented 28.32% (46,273 bp) of the *P. micranthum* plastome, including 12 intact plastome origin genes. Remarkably, the mitogenome of *P. micranthum* and *Gastrodia elata* shared 18% (about 81 kb) of their mitochondrial DNA sequences. Additionally, we found a positive correlation between repeat length and recombination frequency. The mitogenome of *P. micranthum* had more compact and fragmented chromosomes compared to other species with multichromosomal structures. We suggest that repeat-mediated homologous recombination enables the dynamic structure of mitochondrial genomes in Orchidaceae.

## 1. Introduction

The mitochondrion is a key organelle involved in a series of cellular processes. Angiosperm mitochondrial genomes (mitogenomes) are characterized by a low mutation rate, a highly dynamic genome structure, extensive variation in genome size, long non-coding regions, frequent recombination, RNA editing, and widespread horizontal gene transfer [1,2,3,4,5,6,7]. For instance, the smallest mitogenome is found in the hemiparasitic *Viscum scurruloideum*, with a length of 66 kb [8], whereas the mitogenome of *Silene conica* has expanded to 11.3 Mb [9]. Earlier studies have indicated that plant mitogenomes exist as circular structures. However, there is increasing evidence that the genomic conformation of mitogenomes can be more complex than just one circular structure. Electron micrographs of the mitochondria of *Chenopodium album* have shown a subgenome that is circular with a linear tail [10], while some species even show a complex branched structure [11], and a multichromosomal structure has been independently identified in multiple lineages [3,9,11,12,13,14,15,16,17,18,19]. For example, the mitogenome of *S. conica* consists of 128 circular chromosomes ranging in size from 44 kb to 163 kb [9]. The number of mitochondrial chromosomes ranges from 2 in multiple species to 132 in *Picea glauca* [17]. Furthermore, the mitochondrial genes have shown a disparity in their substitution rates [20,21,22], and the synonymous substitution rate in *Ajuga* has shown a 340-fold range [20]. In contrast, *Liriodendron tulipifera* has a “fossilized” mitochondrial genome, which has undergone little change over the last 100 million years [23].

Angiosperm mitogenomes are poorly characterized compared to their plastomes or to animal mitogenomes (NCBI database, 351 land plants mitogenomes, 13 July 2022) and are dominated by species from the crop families, such as Brassicaceae, Fabaceae, Poaceae, and Solanaceae [24]. In addition, the complexity of mitogenome assembly is exacerbated by their multichromosomal structure, recombination, lengthy repeat sequences in intergenic spacer regions and introns, and horizontal gene transfer events [11]. For instance, the intron of *cox2* expanded to 11.4 kb in *Nymphaea colorata*, and the repeat sequences accounted for 49% of its mitogenome [25]. The total length of repeat sequences in *S. conica* even reaches 4621 kb and accounts for 40.8% of the mitogenome [9]. Repeat-mediated homologous recombinations have resulted in different conformations coexisting in the same species [19,25,26,27,28,29,30]. In some cases, the long, plastid-like sequences will lead to erroneous extensions that produce chimeric contigs comprising both mitogenomic and plastomic sequences [31]. With the rapid advances in long-read sequencing technologies and assembly methods, long repeat regions and plastid-derived fragments in the plant mitogenomes [32,33] can now be resolved, which will facilitate the study of angiosperm mitogenomes.

Orchidaceae is one of the largest families of angiosperms, with about 28,000 species (World Checklist of Orchidaceae). All orchids depend on mycorrhizal fungi for seed germination in their initial stage of development, and many orchids rely on mycorrhizal fungi for nutrients in their later life [34,35,36]. The symbiotic relationship between orchids and fungi makes orchids an outstanding candidate for investigating the evolution of mitogenomes. There has only been one draft-assembled mitogenome of Orchidaceae reported to date: the mitogenome of holo-heterotrophic *Gastrodia elata*, consisting of 19 contigs (13.5 kb to 410.3 kb) with a total length of 1340 kb, which is one of the largest mitogenomes of angiosperms sequenced, to date [37]. Further, there are six other orchid species from the subfamily Epidendroideae with multichromosomal structures available in GenBank (unpublished data). The general features of a mitogenome from other clades of Orchidaceae are unknown. Considering the large species number in this family, the mitogenome evolution of orchids needs more case studies. Furthermore, previous studies have shown widespread intracellular gene transfer or horizontal gene transfer, including plastome-to-mitogenome gene transfer, mitogenome-to-nuclear gene transfer, and horizontally transferred foreign sequences in the mitogenome obtained from unrelated plant species [3,12,16,23,31,38,39,40,41,42,43,44,45]. Zhao et al. [38] summarized that the mitochondrion is the source of horizontal gene transfer. For instance, the mitogenome of *Amborella trichopoda* contains entire mitogenomes from three green algae and one moss [3]. Choi et al. [41] identified non-retroviral endogenous RNA viral elements (NERVEs) and transposable elements across legume mitogenomes. Further, the symbiosis between orchids and fungi likely boosts the opportunities for horizontal gene transfer between them. Moreover, Sinn and Barrett [31] found two ancient horizontal transfer events between orchids and fungi: one 270 bp fragment encoding three tRNA genes obtained from the mitogenome of fungi and the other > 8 kb fragment encoding 14 genes from a fungal mitogenome to the mitogenome of ancestors of the subfamily Epidendroideae. Further, Sinn and Barrett [31] speculated that the horizontal events involving plant mitogenomes might be underestimated, owing to the lack of completely sequenced genomes. However, the work of Sinn and Barrett [31] did not cover species from the subfamily Cypripedioideae. 

In this study, we report the sequencing, assembling, and annotation of the mitogenome of *Paphiopedilum micranthum* using a combination of Illumina and PacBio sequencing platforms. *P. micranthum* is a species that was first described in 1951; it is known as the “silver slipper orchid”, has high ornamental value, and is distributed from the north of Vietnam to the southwest of China [46]. We aimed to decipher the structure and gene content of the mitogenome of *P. micranthum* and compare the *P. micranthum* mitogenome with the *G. elata* mitogenome. Furthermore, we wanted to assess the intracellular gene transfer between the plastome and mitogenome of *P. micranthum*, and test the horizontal gene transfer between the mitogenome and fungi detected by Sinn and Barrett [31]. Finally, we calculated the recombination frequency of the repeat pairs and tested the relation between repeat length and the recombination frequency.

## 2. Results

### 2.1. The Multichromosomal Structure of the P. micranthum Mitogenome

**Figure 1:**
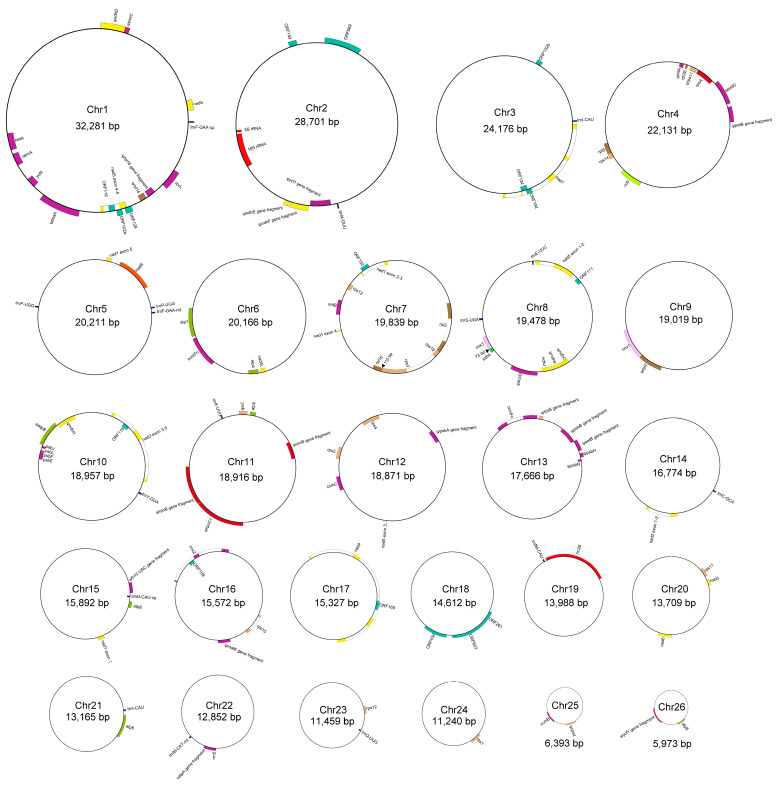
Map of the *Paphiopedilum micranthum* mitogenome. The genome consisted of 26 circular chromosomes. Genes drawn inside and outside each circle are transcribed clockwise and counterclockwise. Two triangles show the positions of the overlapping of *cox3*-*sdh4* and *rpl16*-*rps3*, with the length of overlap indicated.

**Figure 2:**
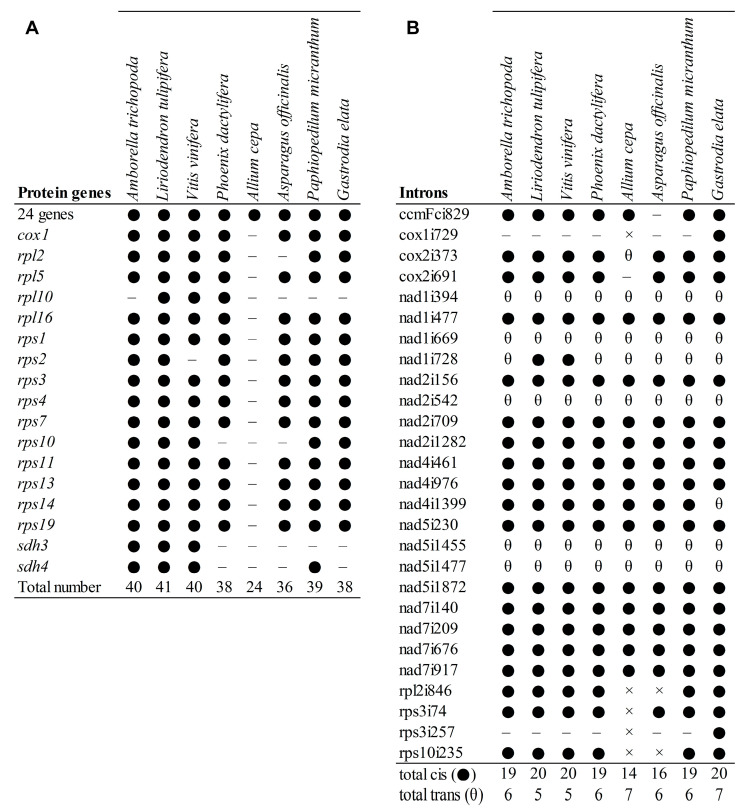
Comparison of the protein-coding gene content and group II intron content of *Paphiopedilum micranthum* and selected angiosperms. (**A**) protein-coding gene content. ● indicates present, – indicates lost; the 24 genes present in all sampled species include *atp1*, *atp4*, *atp6*, *atp8*, *atp9*, *ccmB*, *ccmC*, *ccmFc*, *ccmFn*, *cob*, *cox2*, *cox3*, *matR*, *mttB*, *nad1*, *nad2*, *nad3*, *nad4*, *nad4L*, *nad5*, *nad6*, *nad7*, *nad9*, and *rps12*. (**B**) group II intron content. ● indicates cis-spliced intron present, – intron lost, θ trans-spliced intron present, × intron loss due to gene loss.

The mitogenome of *P. micranthum* was assembled into 26 circular chromosomes with lengths ranging from 5973 bp to 32,281 bp, with a total length of 447,368 bp (Figure 1). The average GC content of the *P. micranthum* mitogenome was 44.4%, ranging between 40.4% and 49.2% among chromosomes (Table 1). We obtained, for most of the chromosomes, a sequencing depth above 40× for the long reads and 500× for the short reads (Table S1). Both long- and short-read assemblies were almost identical, except for Chr5 (20,211 bp), which existed as one circular sequence in the short-read sample and fragmented into Chr5A (9033 bp) and Chr5B (11,178 bp) in the long-read sample. Both minicircles were supported by 32 and 102 long reads, respectively (Figure S1). The mitogenome of *P. micranthum* encoded 70 genes, including 39 mitochondrial protein-coding genes, 12 plastome-derived protein-coding genes, 16 tRNA genes, and three rRNA genes (rrn5, rrn18, and rrn26) (Figure 2, Table 2). Further, 16 ORFs coding for hypothetical proteins with BLAST hits were preserved (Figure 1, Table 2 and Table S2). In addition to the copy of rrn5 on Chr2, which is identical to the one annotated in *G. elata* [37], the copy on Chr22 was truncated at the 5’ end (88 bp) and relatively shorter than the normal one. Each chromosome had one to four genes, whereas Chr18, with a length of 14,612 bp, was devoid of functional genes (Figure 1). Further, the “empty” sequence presented no significant similarities to the sequences in GenBank. 

Most of the mitochondrial genes had a conserved gene length, whereas some genes varied greatly in length, e.g., *atp6* expanded to 1272 bp, *atp9* expanded to 327 bp, *sdh4* contracted to 204 bp (Table S3), and these three genes underwent RNA editing according to the prediction in PREPACT. Furthermore, we identified 25 Group II introns, including 19 cis-spliced introns and six trans-spliced introns, located in seven cis-splicing genes (*ccmFc*, *cox2*, *nad4*, *nad7*, *rpl2*, *rps3*, and *rps10*) and three trans-splicing genes (*nad1*, *nad2*, and *nad5*) (Figure 1, Table 2 and Table S4). The exons of *nad1*, *nad2*, and *nad5* were encoded on different chromosomes (Figure 1). For *nad5*, the five exons of *nad5* separated across three chromosomes, with exon1 and exon2 in Chr8, exon3 in Chr12, and exon4 and exon5 in Chr1 (Figure 1). 

The tRNAs of *P. micranthum* came from different origins, including twelve native mitochondrial-origin tRNAs (*trnE-UUC*, *trnF-GAA*-mt, *trnfM-CAU*, *trnI-CAU*×2, *trnK-UUU*, *trnM-CAU-*mt, *trnP-UGG*×2, *trnQ-UUG*, *trnS-UGA*, and *trnY-GUA*), three plastid-origin tRNAs (*trnF-GAA*-cp, *trnM-CAU*-cp, and *trnN-GUU*), and one bacteria-origin tRNAs (*trnC-GCA*) [42]. The plastome-originating tRNA, *trnF-GAA*-cp, had been reported before in angiosperm mitogenomes [42]. Notably, the mitogenome encoded both *trnF-GAA*-mt (74 bp) and *trnM-CAU-*mt (73 bp) from the mitochondrial origin, and *trnF-GAA*-cp (73 bp) and *trnM-CAU*-cp (73 bp) from the plastome origin. *trnI-CAU* and *trnP-UGG* had duplicated copies in the mitogenome and *trnI-CAU* duplicated in different chromosomes (Chr3 and Chr21), whereas *trnP-UGG* dispersed duplicated in Chr5 (Figure 1). In addition, Chr5 retained a truncated remnant of the plastome-origin *trnV-UAC* (Figure 1). However, some ancient plastome-to-mitogenome tRNAs (e.g., *trnH-GUG* and *trnW-CCA*) that appear in most sequenced angiosperm mitogenomes were not detected, which revealed that the mitogenome of *P. micranthum* experienced gene loss and gain. 

Further, gene synteny analyses indicated that the mitogenome of *P. micranthum* retained eight of the 14 ancestral gene clusters reported across angiosperms [23], including *atp4*-*nad4L*, *rpl2*-*rps19*-*rps3*-*rpl16*, *rpl5*-*rps14*-*cob*, *rps13*-*nad1*.x2.x3, *rrnS*-*rrn5*, *trnfM(CAU)*-*rrnL*, *trnF(GAA)*-*trnP(UGG)*, and *trnY(GUA)*-*nad2*.x3.x4.x5.

### 2.2. Horizontal Gene Transfer or the Intracellular Gene Transfer in the Mitogenome of P. micranthum

**Figure 3:**
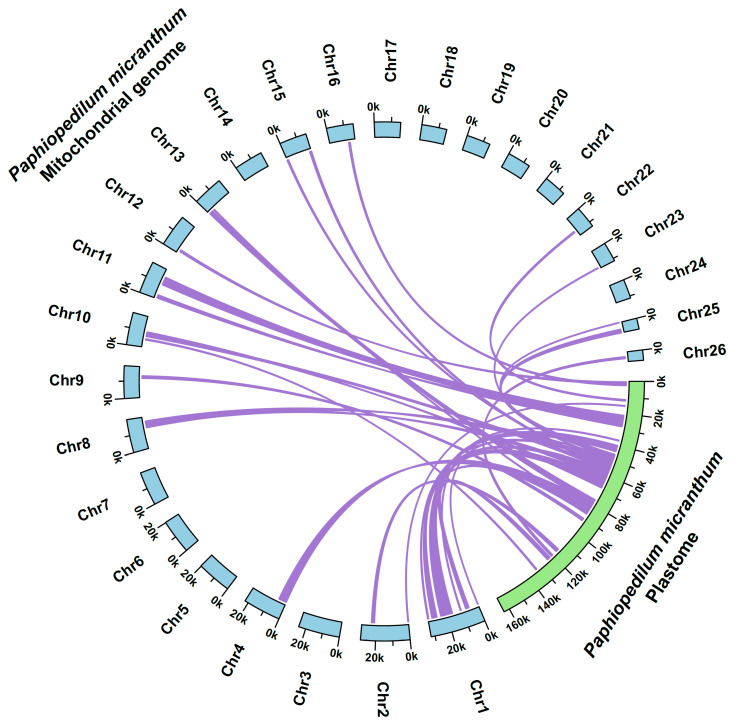
Schematic diagram of gene transfer between the plastome and mitogenome of *Paphiopedilum micranthum*.

A total of 15 of the 26 chromosomes in the mitogenome of *P. micranthum* contained plastome-origin sequences, encoding 12 intact protein-coding genes, 3 tRNAs, and 29 pseudogenes (Table 2 and Table 3). All of these genes were identified by the plastome of *P. micranthum*, except for ψ*ndhE*, ψ*ndhF,* and ψ*ndhH*, which were identified by the plastome of *Cypripedium tibeticum*. However, the 270 bp fungal mitogenomic region identified by Sinn and Barrett [31] was not detected in the mitogenome of *P. micranthum*. The 25 plastome-derived fragments ranged from 165 bp to 7269 bp, with a total size of 46,273 bp, accounting for 10.34% of the whole mitogenome length and 28.32% of the *P. micranthum* plastome (Figure 3, Table 3). Even the smallest chromosome (Chr26—5973 bp) contained a 903 bp plastome-derived sequence. Most of the plastome-derived sequences showed high similarity to their conspecific plastome sequence (ranging from 84.2% to 99.6%) (Table 3), and 12 of the plastome origin genes were intact and potentially functional, including *ycf4*, *cemA*, *petA,* and a shortened *rbcL* (1047 bp) in Chr1; *rpoA* and *rpl36* in Chr4; *ndhJ* in Chr8; *psbE*, *psbF*, *psbJ*, and *psbL* in Chr10; and a shortened *atpE* (345 bp) in Chr15 (Table 2 and Table 3). Particularly, *psbE*, *psbF*, and *trnM-CAU-cp* retained an identical copy with the plastome sequence of *P. micranthum*. However, other plastome-derived genes appeared as pseudogenes, e.g., ψ*ndhD*, ψ*psaA*, ψ*psaC*, and ψ*rpl14* in Chr1; and ψ*atpI*, ψ*ndhE*, ψ*ndhF*, and ψ*ycf1* in Chr2 (Table 2 and Table 3). Further, the chromosomes with plastome-origin fragments (40.4% to 46.3%) had lower GC content compared to chromosomes without plastome-origin sequences (41.2% to 49.2%), e.g., the GC content of Chr17 was 49.2%, while the GC content of Chr25 was 40.4% (Table 2).

### 2.3. Repeat Sequences in the Mitogenome of P. micranthum

**Figure 4:**
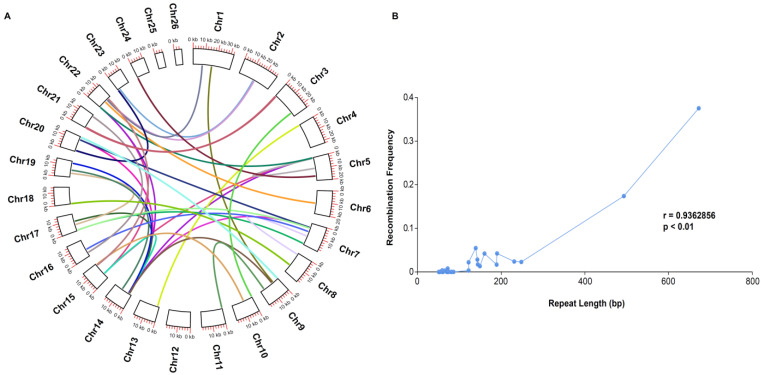
Recombination frequency in the mitogenome of *Paphiopedilum micranthum*. (**A**) The distribution of the 34 pairs of repeats; (**B**) recombination frequency of 34 pairs of repeats (>50 bp) with 100% identity.

Overall, 27 tandem repeats, with lengths ranging from 27 bp to 308 bp, accounted for 1948 bp of the *P. micranthum* mitogenome; these repeats resided in the non-coding regions of the genome, except for a 48 bp repeat in *rrn26*, and some of the tandem repeats overlapped with the dispersed repeats. The mitogenome of *P. micranthum* possessed 89 dispersed repeats (34 types), ranging from 51 bp to 672 bp, with two to four copies and covering 9996 bp (2%) of the genome. The majority of these repeats (87 of 89, 97.7%) were intermediate-sized repeats (50 to 500 bp) and two repeats (672 bp) were large repeats (>500 bp) (Table S5), with most of these repeats residing in the noncoding regions. These repeats were distributed in 23 of the 26 chromosomes; Chr12, Chr25, and Chr26 did not contain dispersed repeats (Figure 4A). We found 16 pairs of repeats involved in the recombination of the mitogenome structure. The alternative conformations were supported by the long reads, and repeat length was positively correlated with the recombination frequency (r = 0.9379, *p* < 0.001), e.g., the recombination frequency of the longest repeat (672 bp) was 0.38; 116 long reads supported the alternative conformations, and 193 long reads supported the master circle conformation, while homologous recombination occurred sporadically among repeats shorter than 300 bp (Figure 4B, Table S5).

## 3. Discussion

### 3.1. General Features of the P. micranthum Mitogenome

The mitogenome of *P. micranthum* was conserved in gene number and gene content compared to other angiosperm mitogenomes, encoding for 39 of the 41 protein-coding genes present in the common ancestors of angiosperms [47]—except for *sdh3* and *rpl10*, which were lost from the mitogenome of *P. micranthum* (Figure 2). *Sdh3* and *sdh4* encoded succinate dehydrogenase, and the two genes had been lost repeatedly in the mitogenomes of angiosperm [39]. While, in many other angiosperm lineages, both *sdh3* and *sdh4* are lost from the mitogenome, *sdh4* was retained in the *P. micranthum* mitogenome. Notably, the *sdh4* in *P. micranthum* contracted to 204 bp; the contraction of *sdh4* was also observed in coconut palm (183 bp) [48], and we annotated a 222 bp of *sdh4* in *Asparagus officinalis* (MT483994). *Rpl10* has frequently been reported as lost in angiosperms [24] and pseudogenized or lost in sequenced monocots [49]. 

**Figure 5:**
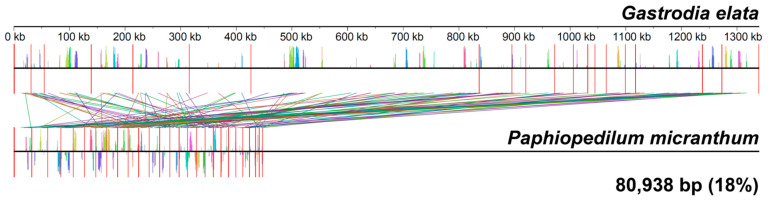
The colinear analysis of the mitogenome of *Paphiopedilum micranthum* and *Gastrodia elata*.

The mitogenome of *P. micranthum* and *G. elata* shared 18% (about 81 kb) of their mitochondrial DNA (mtDNA) sequences, including 37 protein-coding genes (33, 817 bp), and 47, 121 bp non-coding regions (Figure 5). The amount of shared mtDNA was relatively small compared to most other pairs of species in seed plants [50,51]. Compared to the mitogenome of *G. elata*, the gene content of the two species was quite similar, and even the length of the cis-splicing introns was similar (Table S4). The GC content of the 26 chromosomes of *P. micranthum* was more variable compared to the 19 chromosomes of *G. elata.* Owing to active recombination, the ancestral gene clusters conserved across angiosperms were lost in *P. micranthum*; only 8 of the 14 gene clusters conserved across angiosperms were preserved. Even the two gene clusters, *nad9*-*trnY(GUA)* and *trnI(CAU)*-*trnD(GUC)*, restricted to monocots, broke in the *P. micranthum* mitogenome. Further, there were eight gene clusters shared between the mitogenome of *P. micranthum* and *G. elata*, including *atp4*-*ndh4L*, *rpl2*-*rps19*-*rps3*-*rpl16*, *rpl5*-*rps14*-*cob*, *rps13*-*nad1*.x2.x3, *trnY(GUA)*-*nad2*.*x*3.x4.x5, *matR*-*nad1.x1*, *atp1*-*ccmFn*, and *apt9*-*rps7*; the first five of them were conserved in most angiosperms, the other three were newly formed, and *atp1*-*ccmFn* was restricted to Orchidaceae. 

Five of the six trans-splicing introns (nad1i394, nad1i669, nad2i542, nad5i1455, and nad5i1477) were shared with the common ancestors of seed plants [24]. The trans-splicing of nad1i728 (the fourth intron) was sporadically distributed among angiosperms [24,52], and most of the species sequenced in monocots presented trans-splicing of this intron, e.g., *Allium cepa* [53] and *A. officinalis* [54], which indicates rampant recombination in the mitogenome evolution. In the *P. micranthum* mitogenome, the trans-splicing of nad1i728 was owed to the chromosome fragmentation, and exon4 and exon5 of nad1 were located in Chr7 and Chr5, respectively. Guo et al. [52] indicated that cis- shift to trans-splicing correlated with the rearrangement in the seed plants. The intrachromosomal trans-splicing to interchromosomal trans-splicing also indicates active recombination in the mitogenome.

### 3.2. Rampant Plastome Origin Sequences in the Mitogenome of P. micranthum

The plastome origin sequences accounted for 0.1% to 10.3% of the mitochondrial genome [47,55]. For instance, the plastome-derived sequence accounted for 1.98% (11,281 bp) in *Hibiscus cannabinus* [56], 1.16% (8937 bp) to 4.05% (37,483 bp) in kiwifruit [57], 6% (23 kb) in *Citrullus lanatus* [58], and 8.8% in vitis [59]. Further, the plastome-obtained sequences covered less than 5% of most angiosperm mitogenomes [55]. In contrast, 15 of the 26 chromosomes in the *P. micranthum* mitogenome contained plastome-derived fragments, covering 10.34% (~46 kb) of the *P. micranthum* mitogenome (Figure 3, Table 3). Compared to most other reported plastome-derived mitogenome fragments [44,60,61], the plastome-derived sequences in the mitogenome of *P. micranthum* were more pervasive and widespread, both in relative and absolute terms. The plastome-derived sequences in Chr1 even reached 12 kb (38% of Chr1) (Table 3). More than half (27,420 bp, 59%) of the plastome-origin mitogenome sequences were identical to the plastome of *P. micranthum*, with a range in size from 50 to 676 bp (Table 3). These properties suggest that the plastome-derived sequences stem from multiple transfer events, and following their intracellular transfer, these sequences experience multi-rounds of recombination.

Interestingly, *ndh* genes experienced different extents of degradation in the plastome of *Paphiopedilum*, and *ndhE, ndhF*, and *ndhH* have been lost from the plastome of *P. micranthum* [62]. However, the pseudo copies of ψ*ndhE,* ψ*ndhF*, and ψ*ndhH* were detected in *P. micranthum* mitogenome (Figure 1, Table 2 and Table 3). Additionally, *ndhJ* was reported as a pseudogene in the plastome of *P. micranthum* owing to the non-triplet, insertion-induced, premature-stop codons [62], whereas the mitogenome of *P. micranthum* encoded the potential functional copy of *ndhJ*. These data suggest that the transfer events of *ndh* genes predated the degradation of *ndh* in the *P. micranthum* plastome, or there was more than one donor of their plastome origin sequences. Furthermore, most of the plastome origin genes have been nonfunctional pseudogenes in previous studies [3,4,40,42], except for a few cases—for instance, *psaA*, *ndhB*, and *rps7* in *H. cannabinus* [56] and *petN*, *psaA*, *atpI*, *trnI-CAU*, and *trnC-GCA* in *Mangifera* [63]. The mitogenome of *P. micranthum* contained 44 genes from plastid origin, and 12 of these genes are intact and potentially functional (*ycf4*, *cemA*, *petA*, *rbcL*, *rpoA*, *rpl36*, *ndhJ*, *psbE*, *psbF*, *psbJ*, *psbL*, and *atpE*), which has been rather rare in previous studies (Table 2 and Table 3). 

### 3.3. The Multichromosomal Mitogenome Structure of P. micranthum

The mitogenome of *P. micranthum* fragmented into 26 minicircular genomes (5973 bp to 32,281 bp). The mitogenome size of other species with multichromosomal structures varied widely, from 66 kb in *V. scurruloideum* [8] to 11,318 kb in *S. conica* [9], and most of these angiosperm species had two to five contigs [17], except *Cynomorium* [64] *Fagopyrum esculentum* [65], *G. elata* [37], *Geranium brycei* [40], *Lophophytum mirabile* [16], *Ombrophytum subterraneum* [12], *Rhopalocnemis phalloides* [19], and *Silene* [9,66] (Table S6). Compared to most other species with multichromosomal structures, the mitogenome of *P. micranthum* showed a more compact and fragmented genome structure. Notably, the shortest mitogenome of *P. micranthum* was 5973 bp encoding *atp8* and partially ψ*ycf1*; the size of the smallest chromosome was similar to the other species with multichromosomal structures, e.g., *O. subterraneum* (4900 bp) [12] and *R. phalloides* (4949 bp) [19]. The mitogenome fragmentation may facilitate the recombination between physically unlinked loci [66,67]. Further, 9 of the 26 chromosomes were autonomous chromosomes; these chromosomes did not contain repeats longer than 100 bp, and all the autonomous chromosomes in *P. micranthum* contained protein-coding genes, while the autonomous chromosomes in cucumber and *Silene* do not contain identifiable genes [9,15].

Repeat sequences are a source of constant rearrangement in the mitogenome [14,68,69,70]. Direct repeat-mediated recombination has been documented in previous studies, e.g., *Brassica campestris* [71] and *Scutellaria tsinyunensis* [27]. Li et al. [27] reported a pair of direct repeats (175 bp) mediated recombination in the mitogenome of *S. tsinyunensis*, and the 354,073 bp master circle was fragmented into two chromosomes with a length of 255,741 bp and 98,402 bp. According to the conventional multipartite model, large repeats in the master circle induced intragenomic recombination, resulting in a set of subgenomic circles [14]. Chr5 was fragmented into Chr5A and Chr5B due to a pair of 122 bp repeats (Figure S1). However, we did not detect the master circle that included the entire sequence of the *P. micranthum* mitogenome. Notably, *P. micranthum* had fewer repeat sequences, both in number and relative percentage compared to other monocot species tested [5]. Further, the number of repeat sequences (2%) was relatively less than most other species, e.g., *Mangifera* (3.5% to 4.5%) [63], *Monsonia* (3.9% to 6.9%) [68], *Trifolium* (6.6% to 8.6%) [72], and *Silene vulgaris* (18.8 to 28%) [69]. 

Long-read sequencing provided a reliable method to explore repeat-mediated homologous recombination. Though the mitogenome of *P. micranthum* does not contain repeats longer than 1 kb, we found alternative conformations that coexisted in the flanking regions of repeat sequences. Further, the repeat length was strongly correlated with the recombination frequency (r = 0.9379) (Figure 4, Table S5), which has also been identified in previous studies [8,9,73]. The mitogenome of *P. micranthum* was depicted as 26 minicircles for simplicity. In fact, long-read sequencing implied that the mitogenome of *P. micranthum* consisted of a population of alternative structures that resulted from dispersed repeats. Moreover, repeat sequences might play an important role in the mitogenome fragmentation of Orchidaceae.

## 4. Materials and Methods

### 4.1. Genome Sequencing, Assembly, and Annotation

We collected two fresh leaf samples of *P. micranthum* from the National Orchid Conservation and Research Center of Shenzhen (NOCC). Total genomic DNA was extracted using the cetyltrimethyl ammonium bromide (CTAB) method [74]. The extracted total genomic DNA was used for library construction with 350 bp and 20 kb insert sizes and then sequenced on MGI2000 (MGI, Shenzhen, China) and PacBio RS-II platforms (Pacific Biosciences, Menlo Park, CA, USA) for short and long reads, respectively.

The long reads were error-corrected using Canu v2.0 [75]. Then, we used the mitogenome sequences, downloaded from GenBank, as reference sequences. The potential mitogenome long reads were filtered with BLASR v5.1 [76]; short reads were filtered with a perl script described in Wang, et al. [77], and the enriched reads were used for hybrid assembly in SPAdes v3.14.1 [78]. Mitogenome contigs were filtered using BLASTN [79] and used as reference sequences for further analysis. We repeated the above steps for multiple rounds in SPAdes to improve the assembly.

In parallel, we used the complete, uncorrected datasets to assemble the mitogenome with an unpublished hybrid assembly version of NOVOPlasty [80,81]. As a seed-and-extend assembler, it needs a mitochondrial seed to initiate the assembly. Since this mitogenome exists out of multiple circular genomes, we selected all the protein-coding genes shared among angiosperms as seed sequences. Furthermore, we used the mitochondrial contigs from the SPAdes assembly that were devoid of genes as additional seeds. The overlapped regions of the above-described methods are identical, and contigs obtained from SPAdes usually lost some parts of the circular genome. In addition, we mapped the long PacBio reads to these contigs to verify the results and we de novo assembled the lost genes (*rpl10* and *sdh3*) with NOVOPlasty to confirm their absence. 

The assembled contigs were annotated in Geneious Prime (Biomatters, Inc., Auckland, New Zealand) with *L. tulipifera* [23], *G. elata* [37], *A. cepa* [53], and *A. officinalis as references* [54], and refined manually. Open reading frames (ORFs) were predicted and annotated using ORFfinder in Geneious Prime, starting with ATG and of length > 300 bp. tRNA genes were annotated using tRNAscan-SE v2.0 [82]. The obtained contigs were deposited in GenBank under accession numbers OP465200–OP465225 (Table 1). The genome maps were generated with OrganellarGenomeDRAW [83]. Additionally, we used PREPACT 3.12.0 [84] to predict RNA editing in three genes (*atp6*, *atp9*, and *sdh4*) reference to *Amborella* and *Liriodendron*.

### 4.2. Identification of Plastid-Derived Regions and Other Horizontally Derived Regions

Firstly, the mitogenome of *P. micranthum* was searched against the plastomes of *P. micranthum* (MN587791) [62] and *C. tibeticum* (MT937101) [85] to identify plastid-derived fragments with BLAST v2.11.0+ [79], using a word size of seven, an E-value cutoff of 1 × 10^−6^, and a length > 100 bp. The paralogs in the mitogenomes and plastomes were excluded from the results (e.g., *atp*1/*atp*A, *rrn*26/*rrn*23, and *rrn*18/*rrn*16), following the procedures in Guo et al. [51]. Additionally, we compared the mitochondrial homologs with putative plastid regions to evaluate the mutations in the plastid-derived mitochondrial genes. Then, we use the mitogenome of *Ustilago maydis* as reference sequences to identify the horizontal gene transfer fragments mentioned in Sinn and Barrett [31].

### 4.3. Repeat and Repeats-Mediated Homologous Recombinations

Tandem repeats in the *P. micranthum* mitogenome were identified using Tandem Repeat Finder v4.09 [86] with default parameters. The dispersed repeats were detected using the python tool ROUSFinder.py [5] with a minimum repeat size of 50 bp. Then, we calculated the recombination frequency of 34 pairs of repeats with 100% identity, following the methods of Sullivan et al. [70]. For each repeat pair, we extracted ±2000 bp flanking regions and constructed two potentially alternative conformations. The recombination rate was calculated by dividing the number of recombinant reads by the total number of reads spanning each repeat. In addition, we tested whether the repeat length correlated with the recombination frequency.

## 5. Conclusions

We accurately assembled the mitogenome of *P. micranthum* with a combination of long- and short-read data. The mitogenome of *P. micranthum* presents typical multichromosomal structures and preserves a large amount of plastome-derived horizontal gene transfer fragments. Considering the genome size and chromosome number, the mitogenome of *P. micranthum* is more fragmented than most other species with multichromosomal genome structures. The long reads provide strong evidence for the plastome-to-mitogenome intracellular gene transfer and the repeat-mediated homologous recombination. The comparison of the *P. micranthum* mitogenome with the mitogenome of *G. elata* sheds light on the mitogenome evolution of Orchidaceae. Though the mitogenomes of the two species have similar gene content, the mitogenomes of the two species share only 81 kb of their mtDNA sequence. Considering the disparities in genome size and chromosome number, the high frequency of recombination, intraspecies genome structure variation, and the low collinearity of the two orchids, our understanding of the mitogenome evolution of orchids is rather limited. Further studies are needed to unravel the mitogenome evolution of orchids.

## Figures and Tables

**Table 1:** General features of the mitogenome of *Paphiopedilum micranthum*.

Genome Feature	*Paphiopedilum micranthum*
Genome size (bp)	447,368
Numbers of contigs	26
Contig length	5973 to 32,281
GenBank Nos	OP465200–OP465225
GC content (%)	40.4% to 49.2%
Length of the coding region (%)	40,029 (8.95%)
Length of rRNA genes (%)	5563 (1.24%)
Length of tRNA genes (%)	1206 (0.27%)
Length of cis-spliced introns (%)	27,985 (6.26%)
length of the plastid-derived sequence (%)	46,273 (10.34%)
Number of protein-coding genes (native)	39
Number of protein-coding genes (plastid derived)	12
Number of rRNA genes	3
Number of tRNA genes (native)	13
Number of tRNA genes (plastid derived)	3
Total genes	70

**Table 2:** Gene content of the mitogenome of *Paphiopedilum micranthum*.

Chromosome	Length (bp)	GC Content (%)	Genes of Mitochondrial Origin	Genes of Chloroplast Origin	ORF
Chr1	32,281	42.2	*nad5* exon 4, *nad5* exon 5, *nad9*	*cemA*, ψ*ndhD*, *petA*, ψ*psaA*, ψ*psaC*, *rbcL*, ψ*rpl14*, ψ*rpl16* fragment, *ycf4*, *trnF*-*GAA*-cp	ORF102a, ORF116, ORF128
Chr2	28,701	43.1	*rrn5*, *rrn18*	ψ*ndhE* fragment, ψ*ndhF* fragment, ψ*ycf1* fragment, *trnN*-*GUU*	ORF149, ORF669
Chr3	24,176	46.8	*nad7*, *trnI*-*CAU*	—	ORF102b, ORF104, ORF124
Chr4	22,131	42.7	*cob*, *rpl5*, *rps14*	ψ*petB* fragment, ψ*petD* fragment, *rpoA*, ψ*rps11*, *rpl36*, ψ*infA*	
Chr5	20,211	45.3	*matR*, *nad1* exon 5, *trnF*-*GAA*-mt, *trnP*-*UGG* (2)	—	
Chr6	20,166	43.1	*atp1*, *atp4*, *ccmFn*, *nad4L*	—	
Chr7	19,839	47.2	*mttB*, *rpl2*, *rpl16*, *rps3*, *rps13*, *rps19*, *nad1* exon 2, *nad1* exon 3, *nad*1 exon 4	—	ORF152
Chr8	19,478	43.1	*nad5* exon1, *nad5* exon2, *trnE*-*UUC*, *trnS*-*UGA*, *cox3*, *sdh4*	ψ*accD*, *ndhJ*, ψ*ndhK*, ψ*ndhC*	ORF111
Chr9	19,019	45.8	*cox1*	ψ*rpl2*	
Chr10	18,957	46.1	*nad2* exon 3, *nad2* exon 4, *nad2* exon 5, *trnY*-*GUA*	ψ*ndhH*, ψ*atpB*, *psbJ*, *psbL*, *psbF*, *psbE*	ORF119
Chr11	18,916	42.2	*atp9*, *rps7*, *trnK*-*UUU*	ψ*rpoB* fragment, ψ*rpoC1*	
Chr12	18,871	43.9	*ccmC*, *rps2*, *rps4*, *nad5* exon3	ψ*psbA* fragment	
Chr13	17,666	44.6	*ccmFc*	ψ*psbN*, ψ*psbH*, ψ*petB* fragment, ψ*psbB* fragment, ψ*rps8* fragment	
Chr14	16,774	44.9	*nad2* exon1, *nad2* exon2, *trnC*-*GCA*	—	
Chr15	15,892	43.3	*nad1* exon1	*atpE*, ψ*trnV*-*UAC* fragment, *trnM*-*CAU*-cp	
Chr16	15,572	46.1	*cox2*, *rps10*	ψ*matK* fragment	ORF109
Chr17	15,327	49.2	*nad4*	—	ORF165
Chr18	14,612	41.2	—	—	ORF261, ORF432, ORF603
Chr19	13,988	45.5	*rrn26*, *trnfM*-*CAU*-mt	—	
Chr20	13,709	45.8	*nad3*, *nad6*, *rps11*	—	
Chr21	13,165	45.4	*atp6*, *trnI*-*CAU*	—	
Chr22	12,852	46.3	*rrn5* fragment, *trnM*-*CAU*	ψ*atpA* fragment	
Chr23	11,459	43.2	*rps12*, *trnQ*-*UUG*	—	
Chr24	11,240	44.9	*rps1*	—	
Chr25	6393	40.4	*ccmB*	ψ*rps4*	
Chr26	5973	42.2	*atp8*	ψ*ycf1* fragment	

**Table 3:** Plastid-derived regions in the mitochondrial genome of Paphiopedilum micranthum.

Chromosome	Length (bp)	Position	Genes Contained	Identity (%)
Chr1	1862	6022–7883	ψ*psaC*–ψ*ndhD*	95.50
Chr1	256	11,166–11,421	none	99.60
Chr1	7269	16,713–23,981	*petA*–*cemA*–*ycf4*–ψ*psaA*	95.90
Chr1	1024	26,693–27,716	ψ*rpl14*–ψ*rpl16*	88.50
Chr1	1549	28,034–29,582	*rbcL*	95.20
Chr1	426	(31,892–32,281) + (1–36)	*trnF(GAA)*	85.20
Chr2	165	5–169	none	94.80
Chr2	1357	19,699–21,055	ψ*ndhE* fragment–ψ*ndhF* fragment	93.00
Chr2	1583	21,146–22,728	ψ*ycf1* fragment–*trnN(GUU)*	91.50
Chr4	4719	157–4875	ψ*petB* fragment–ψ*petD* fragment–*rpoA*–ψ*rps11*–*rpl36*–ψ*infA*	92.30
Chr8	3976	12,854–16,829	ψ*accD*–*ndhJ*–ψ*ndhK*–ψ*ndhC*	93.80
Chr9	1320	11,951–13,270	ψ*rpl2*	96.40
Chr10	415	4673–5087	none	90.90
Chr10	4310	5152–9461	ψ*ndhH*–ψ*atpB*–*psbJ*–*psbL*–*psbF*–*psbE*	93.60
Chr11	1749	566–2314	ψ*rpoB* fragment	87.40
Chr11	4823	9374–14,196	ψ*rpoB* fragment–ψ*rpoC1*	92.60
Chr12	947	1515–2461	ψ*psbA* fragment	92.20
Chr13	2179	400–2578	ψ*psbN*–ψ*psbH*–ψ*petB* fragment–ψ*psbB* fragment	92.50
Chr13	230	3792–4021	ψ*rps8* fragment	88.30
Chr15	1552	(14,959–15,892) + (1–618)	*atpE*–*trnM(CAU)*–ψ*trnV(UAC)* fragment	90.90
Chr16	729	11,631–12,359	ψ*matK* fragment	84.20
Chr22	587	8861–9447	ψ*atpA* fragment	88.30
Chr23	203	10,708–10,910	none	85.80
Chr25	2140	(4328–6393) + (1–74)	ψ*rps4*	84.20
Chr26	903	3032–3934	ψ*ycf1* fragment	89.40

## Data Availability

The annotated mitogenomes generated in this study are deposited in GenBank under accession Nos. OP465200–OP465225.

## Supplementary Materials

The following are available online at https://www.mdpi.com/article/10.3390/ijms24043976/s1.
