# Supplementary material for: Multichromosomal Mitochondrial Genome of Paphiopedilum micranthum: Compact and Fragmented Genome, and Rampant Intracellular Gene Transfer
Source: Int J Mol Sci. 2023 Feb 16;24(4):3976. doi: 10.3390/ijms24043976 (PMC9966765; doi:10.3390/ijms24043976)
Supplement: Supplementary file 1 [file ijms-24-03976-s001.zip › Figure S1 MT_Chr5.pdf]

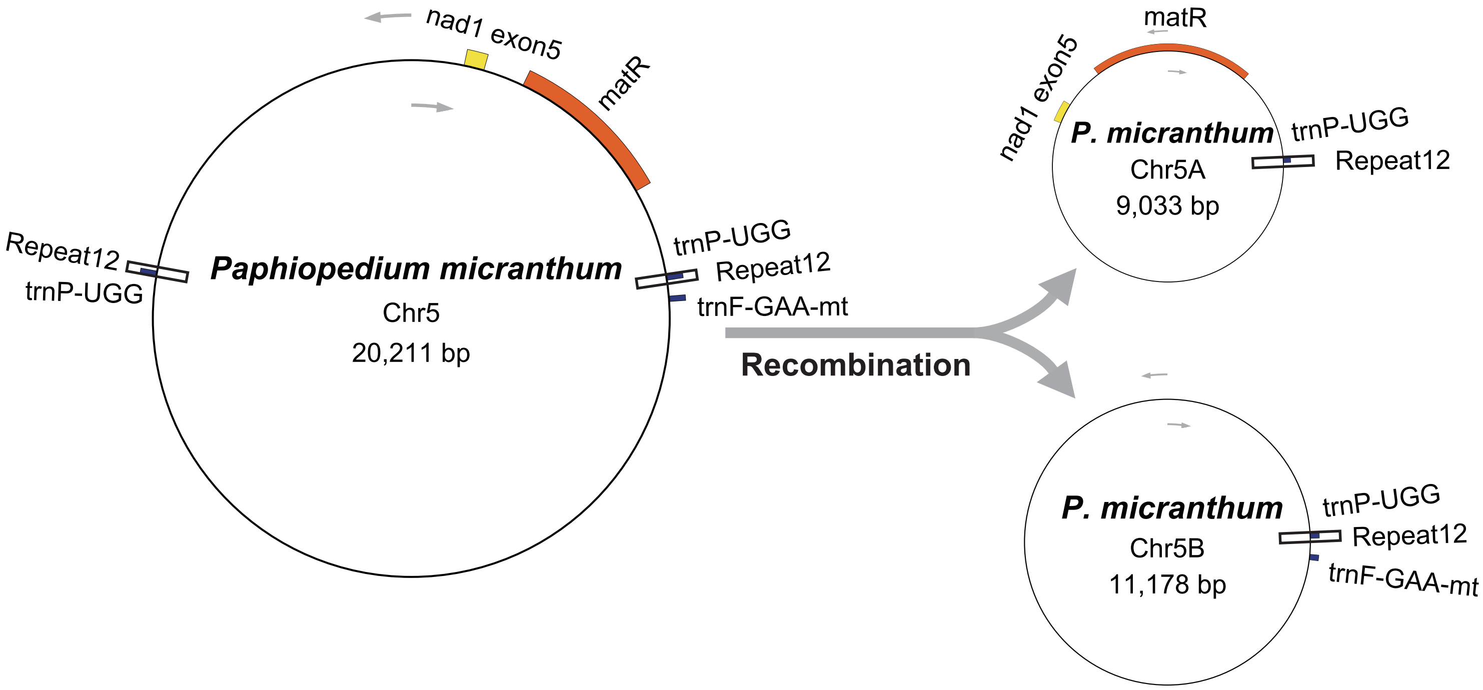

Figure S1. Putative circular map of two conformations of *Paphiopedium micranthum* mitogenome Chr5.
